# Time of day and sleep effects on motor acquisition and consolidation

**DOI:** 10.1038/s41539-023-00176-9

**Published:** 2023-09-01

**Authors:** Charlène Truong, Célia Ruffino, Jérémie Gaveau, Olivier White, Pauline M. Hilt, Charalambos Papaxanthis

**Affiliations:** 1 INSERM UMR1093-CAPS, Université Bourgogne Franche-Comté, UFR des Sciences du Sport, F-21000 Dijon, France; 2 EA4660, C3S Laboratory, C3S Culture Sport Health Society, Université de Bourgogne Franche-Comté, UPFR Sports, 25000 Besançon, France; 3 Pôle Recherche et Santé Publique, CHU Dijon Bourgogne, F-21000 Dijon, France

**Keywords:** Consolidation, Forgetting

## Abstract

We investigated the influence of the time-of-day and sleep on skill acquisition (i.e., skill improvement immediately after a training-session) and consolidation (i.e., skill retention after a time interval including sleep). Three groups were trained at 10 a.m. (G10_am_), 3 p.m. (G3_pm_), or 8 p.m. (G8_pm_) on a finger-tapping task. We recorded the skill (i.e., the ratio between movement duration and accuracy) before and immediately after the training to evaluate acquisition, and after 24 h to measure consolidation. We did not observe any difference in acquisition according to the time of the day. Interestingly, we found a performance improvement 24 h after the evening training (G8_pm_), while the morning (G10_am_) and the afternoon (G3_pm_) groups deteriorated and stabilized their performance, respectively. Furthermore, two control experiments (G8_awake_ and G8_sleep_) supported the idea that a night of sleep contributes to the skill consolidation of the evening group. These results show a consolidation when the training is carried out in the evening, close to sleep, and forgetting when the training is carried out in the morning, away from sleep. This finding may have an important impact on the planning of training programs in sports, clinical, or experimental domains.

## Introduction

Motor learning is essential for the development of new motor skills and the perfection or preservation of existing ones^1^. Practice is fundamental to the acquisition of a profuse motor repertoire. The positive effects of practice on skill acquisition can be observed through different timescales, from a single session to several days, weeks, or months^2^. Typically, a single practice session leads to a rapid increase in accuracy and/or speed until one attains an asymptotic level of performance^3^. This fast learning process, defined as *motor acquisition*, is the first step to the formation of new motor memories. With additional practice sessions and during the rest period between them, a consolidation process transforms the new initially labile motor memory into a robust motor memory (for instance, the performance in a motor sequence enhances after a period of rest, defined as off-line learning)^4,5^. Notably, several studies, including diurnal and nocturnal sleep groups, have highlighted the key role of sleep on motor skill consolidation^6–9^. Both acquisition and consolidation processes are associated with neural adaptations and plastic modulations at several levels of the central nervous system^2^.

Optimization of skill learning is important for many activities and the literature on this topic is abundant. For example, the amount of training^10,11^, the distribution of rest periods during a training session^12,13^, or the variation of skills within a single training session^14,15^ have been the topic of dedicated investigations. Astonishingly, although several studies have shown that motor and mental performances fluctuate across the day, following a circadian basis (∼24 h)^16–18^, the search for the optimal time-of-day for motor learning has not yet retained great attention. Daily variations were observed for maximal voluntary contractions^19^, spontaneous motor tempo^20^, speed/accuracy tradeoff of actual and mental movements^21^, handwriting^22^, and tennis service^23^. In all studies, better performances in strength or skill were consistently reported in the late afternoon than early in the morning.

Sporadic and contradictory information regarding the time-of-day influence on motor learning can be found in the literature. For example, some investigations did not find differences in skill learning between morning and evening training^24–27^. Others tempered this finding by showing that the time-of-day positively affects the expression of motor learning^28–30^. These studies, however, specifically focused on the role of sleep in motor learning, and therefore they cannot provide a direct and compelling investigation of the time-of-day effect on motor learning.

Here, we designed a specific experimental protocol to explore the influence of time-of-day in skill acquisition and consolidation. In the main experiment, we tested three groups that practiced at different times-of-day: 10 a.m., 3 p.m., and 8 p.m., on a finger-tapping task. We measured skill acquisition, namely the enhancement in skill performance during and immediately after practice, and skill consolidation, i.e., the enhancement in skill performance 24 h later. Following the time-of-day literature^16^, we hypothesized that skill acquisition and consolidation should be better in the afternoon compared to the morning. We also motivated this premise by neurophysiological findings showing that physiological mechanisms are modulated throughout the day, such as the cortisol diurnal secretion related to LTP-like plasticity in the motor cortex^31^ and the degree of hippocampus activation^32,33^, associated with consolidation^34,35^. We also designed two control experiments to specifically elucidate the effects of sleep on motor skill consolidation.

## Results

### Main experiment

**Fig. 1:**
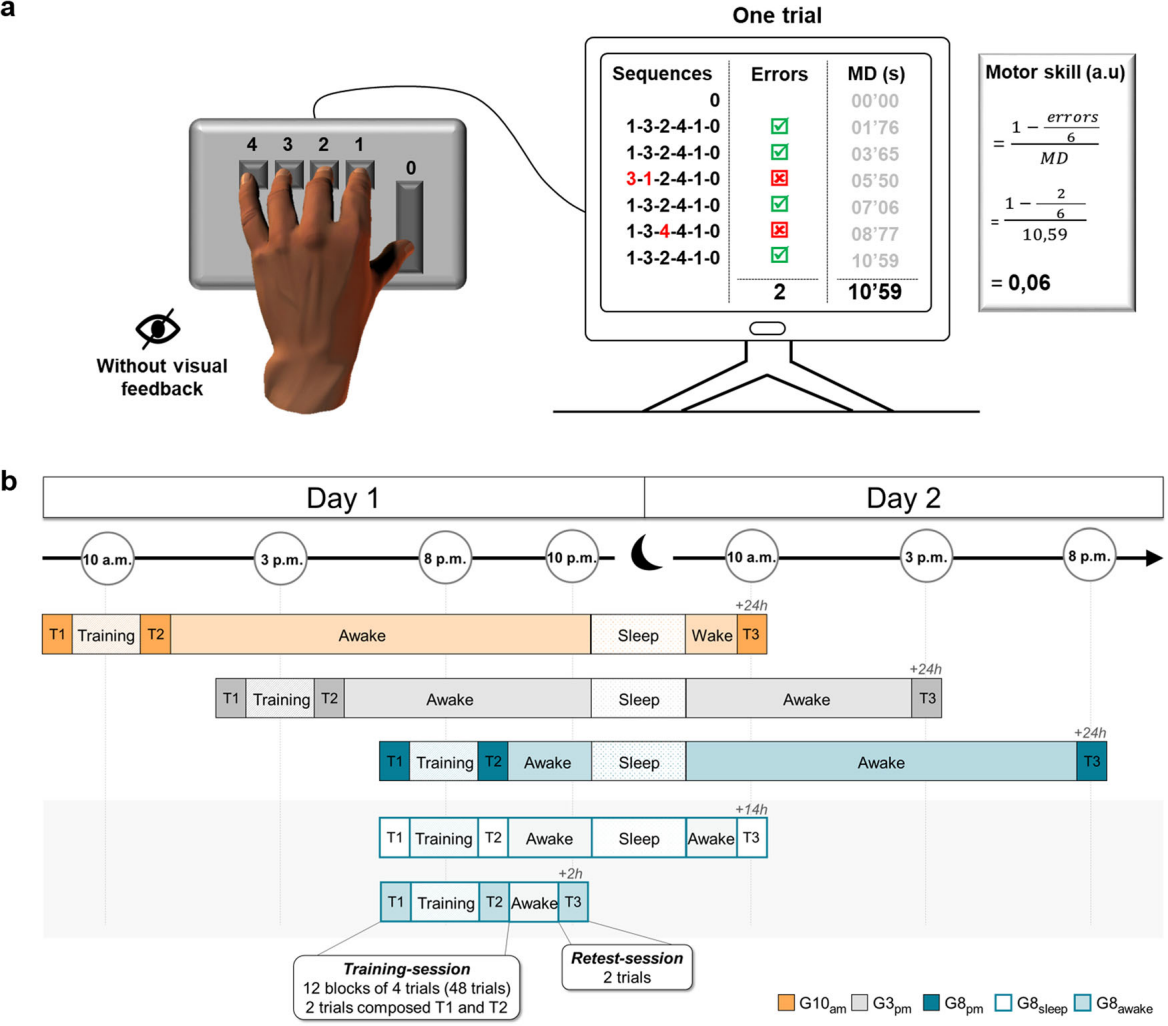
Experimental design. **a** Participants’ hand position on the keyboard and the computerized version of the sequential finger-tapping task. Each key was assigned to a specific finger: 0-thumb, 1-index, 2-middle, 3-ring, and 4-little. One trial included 6 successive sequences: 1 – 4 – 2 – 3 – 1 – 0. At the beginning of each trial, participants pressed the key ‘0’ with their thumb to start the chronometer and they accomplished the 6 sequences in succession. Pressing the key ‘0’ at the end of the 6^th^ sequence stopped the chronometer and ended the trial. We recorded the number of false sequences and the duration of the whole trial (MD). **b** Experimental procedure. On Day 1, participants performed a training-session at 10 a.m. (G10_am_), 3 p.m. (G3_pm_), and 8 p.m. (G8_pm_, G8_sleep_, G8_awake_). All participants carried out 48 trials (12 blocks of 4 trials, with 5-s rest between trials and 30-s rest between blocks). The pre-test (T1) and the post-test (T2) were composed, respectively, of the first two trials (1 and 2) and the last two trials (47 and 48). Then, for the retest-session, all participants accomplished two supplementary trials (T3). The G10_am_, G3_pm,_ and G8_pm_ were tested 24 h after the training (Day 2). The G8_sleep_ and the G8_awake_ were tested 14 h after the training (Day 2 at 10 a.m.) and 2 h after the training (Day 1 at 10 p.m.), respectively.

Thirty-six healthy adults, all right-handed, performed a finger sequential tapping task using their non-dominant hand on a computer keyboard (Fig. 1a). The participants were randomly divided into three groups: the G10_am_ (trained at 10 a.m.), the G3_pm_ (trained at 3 p.m.) and the G8_pm_ (trained at 8 p.m.). We evaluated the acquisition process during the training on day 1 (i.e., the improvement in motor skill), with the initial two trials (1 and 2, pre-test, T1), as well as the final two trials (47 and 48, post-test, T2). The remaining trials (3–46, *n* = 44) constituted the training phase. To measure the consolidation process, all participants carried out two additional trials 24 h later on day 2 (T3) (Fig. 1b). Throughout the experiment, we recorded the accuracy and speed of the sequence execution and defined the motor skill as the combination of both (see Fig. 1a).

Hereafter, we provided the main results for the skill parameter. A similar analysis for both speed and precision is reported in Supplementary notes 1.

**Fig. 2:**
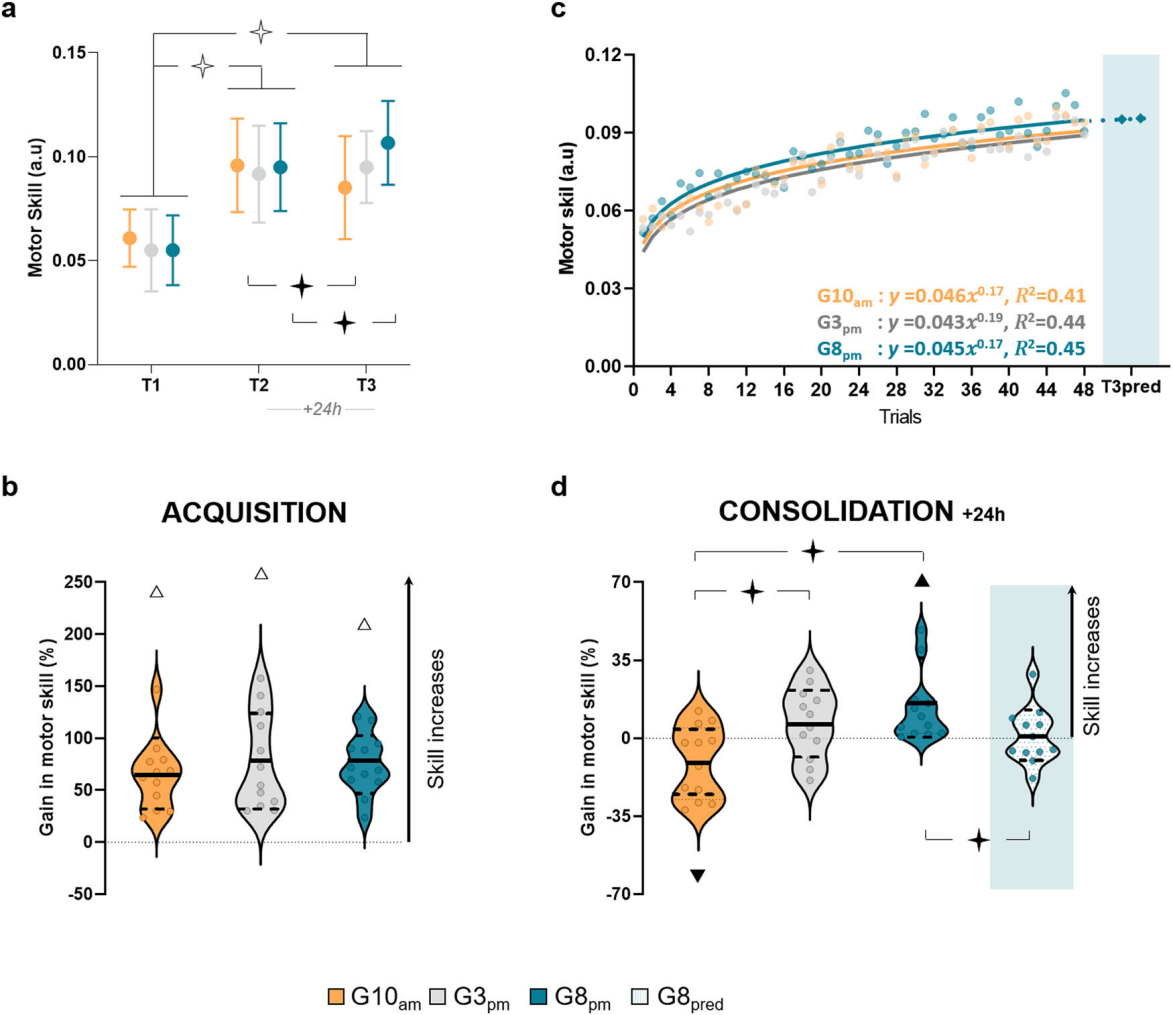
Skill performance for the G10_am_, G3_pm_, and G8_pm_ groups. **a** Average values and standard deviations (+SD) of skill in T1, T2, and T3 for each group. Repeated measures ANOVA and Newman-Keuls post hoc comparisons were applied to the data. **b** Violin plots for the percentage of acquisition gain in skill (T1_T2). Thick and thin horizontal lines mark mean and SD, respectively. Dots represent individual data. No significant difference was found with a one-way ANOVA between groups. **c** Trial-by-trial plotting of skill evaluation during the training. Dots correspond to the group means for each trial and curves correspond to the power law functions using the mean point for each trial. Diamonds are the extrapolation to the two additional trials composing the T3_pred_. **d** Violin plots for the percentage of consolidation gain in skill (T2_T3) for each group and the G8_pred_ (prediction of gain by extrapolation for G8_pm_). Thick and thin horizontal lines mark mean and SD, respectively. Dots represent individual data. A one-way ANOVA and Newman-Keuls post hoc comparisons were applied between G10_am_, G3_pm_, and G8_pm_. Additionally, an independent sample T-test was performed between G8_pm_ and G8_pred_. Stars indicate significant differences between groups or sessions. ✧ *p* < 0.001, ✦ *p* < 0.05. Triangles indicate significant differences from the value zero (*T*-test). Δ *p* < 0.001, ▴ *p* < 0.05.

Figure 2a shows the average values (+SD) of skill for G10_am_, G3_pm,_ and G8_pm_. rmANOVA revealed a main significant effect of the *session* (F_4,66_ = 175.18, *p* < 0.001, η^2^ = 0.84) and an interaction effect (*group* x *session*; F_2,66_ = 5.68, *p* = 0.001, η^2^ = 0.26) but not a main significant effect of *group* (F_2,33_ = 0.42, *p* = 0.66, η^2^ = 0.02). The *post-hoc* analysis did not show any significant difference between groups in T1 (*p* > 0.7 in all cases). In addition, all Bayesian equivalence tests between groups in T1 showed the overlapping hypothesis Bayes Factor (BF^OH^_01_) was superior to 2.45 and the non-overlapping hypothesis Bayes Factor (BF^NOH^_01_) was superior to 2.85, meaning that the data are 2.5 times more likely to validate the null hypothesis than the alternative one and 2.9 times more likely to lie in the equivalence than in the non-equivalence region. Overall, these results suggest that the initial level was comparable between groups.

Skill significantly enhanced after training (T2) for all groups (T1 versus T2 *post-hoc* comparisons: *p* < 0.001 in all cases; see Fig. 2a). Figure 2b illustrates the average (+SD) acquisition gains (T1_T2) in skill. The T1_T2 gain was not significantly different between groups (one-way ANOVA: F_2,33_ = 0.41, *p* = 0.67, η^2^ = 0.02; Bayesian equivalence tests: BF^OH^_01_ > 2.12 and BF^NOH^_01_ > 2.38 in all cases). The comparison with the reference value *zero (0)* confirmed significant improvement for all groups (G10_am_: *t* = 6.71, *p* < 0.001, *d* = 1.94; G3_pm_: *t* = 5.87, *p* < 0.001, *d* = 1.69; G8_pm_: *t* = 9.03, *p* < 0.001, *d* = 2.61).

To further explore acquisition, we examined the skill learning curves adjusted according to power function (Fig. 2c). The analysis did not reveal a significant difference between groups (factor *b*: F_2,33_ = 0.54, *p* = 0.54, η^2^ = 0.04; Bayesian equivalence tests: BF^OH^_01_ > 1.69 and BF^NOH^_01_ > 1.82 in all cases), indicating that the learning rate did not differ according to the time-of-day. Additionally, we did not find any modulation of the moment at which the skill enhancement reached an asymptote (G10_am_: 26 ± 5 trials; G3_pm_: 27 ± 3 trials; G8_pm_: 27 ± 3 trials; F_2,33_ = 0.37, *p* = 0.69, η^2^ = 0.02; Bayesian equivalence tests: BF^OH^_01_ > 2.12 and BF^NOH^_01_ > 2.33 in all cases), whatever the threshold chosen (see also Supplementary notes 2). Overall, the above results indicate that skill improvement during the training sessions does not differ between groups, suggesting that the acquisition process is independent of the time-of-day.

Regarding skill consolidation, we found notable differences between groups, explaining the interaction effect found for skill (*group* x *session*). Precisely, one day after training, there was a deterioration in skill performance for G10_am_ (T2 versus T3; *post-hoc* analysis: *p* = 0.03), a stabilization for G3_pm_ (T2 versus T3; *post-hoc* analysis: *p* = 0.71), and further improvement for G8_pm_ (T2 versus T3; *post-hoc* analysis: *p* = 0.02) (Fig. 2a). Figure 2d illustrates the average (+SD) consolidation gains (T2_T3). One-way ANOVA (F_2,33_ = 7.44, *p* = 0.002, η^2^ = 0.32) showed that T2_T3 gain of the G10_am_ significantly differed from that of the G3_pm_ and G8_pm_ (*p* = 0.01 and *p* = 0.002, respectively; Fig. 2d). Moreover, the comparison of T2_T3 gain with the reference value zero (0) showed a deterioration for the G10_am_ (*t* = −2.27, *p* = 0.04, *d* = 0.66; 9/12 participants decreased their performance), stabilization for the G3_pm_ (t = 1.49, *p* = 0.17, *d* = 0.43; 8/12 participants increased their performance), and an improvement of skill for the G8_pm_ (*t* = 3.02, *p* = 0.01, *d* = 0.87; 12/12 participants increased their performance).

Importantly, skill improvement for the G8_pm_ at T3 was not due to a simple continuation of the practice, namely to the improvement that this group would obtain if two more trials would be carried out on Day 1 (50 trials instead of 48), but rather to offline learning. Indeed, the T2_T3 gain of G8_pm_ was significantly different from the T2_T3_pred_ gain (*t* = 2.21, *p* = 0.04, *d* = 0.90; see Fig. 2c and d). Note that, due to inter-trial variability in T2, we observed negative and positive values in individual T2_T3_pred_ gains. Although the gain results of the G8_pm_ were similarly affected by T2 inter-trial variability, all participants showed positive gains.

Despite group differences in offline skill improvement after training (T2 versus T3), all groups showed better skill performance one-day later (T3) compared to their initial performance (T1 versus T3; *post-hoc* comparisons: *p* < 0.001 in all cases). To exclude any effects of different chronotypes, we conducted the same statistical analyses without the extreme and moderate chronotypes and we obtained the same results (see Supplementary notes 3).

Overall, the consolidation process (i.e., skill measured 24 h later) was affected by the time of day wherein the training was carried out. This finding suggests that physical training scheduled in the evening, close to the night of sleep, may enable offline learning, while that scheduled in the morning may lead to forgetting. Before asserting such a premise, we controlled for alternative issues.

### Control experiment 1

In the main experiment, the groups were retested (T3) at different times on Day 2, to respect a 24 h delay. Precisely, the participants of the G8_pm_ were retested twelve hours after waking up (8 a.m.–8 p.m.), whilst those of the other groups were tested 2 h (G10_am_; 8 a.m.–10 a.m.) or 7 h (G3_pm_; 8 a.m.–3 p.m.) after waking up. Hence, we controlled for a possible effect of the time being awake the day after training (Day 2), which could positively contribute to skill consolidation. The G8_sleep_ performed the training at 8 p.m. and was retested the next day at 10 a.m. (i.e., a total of 14 h after the training but 2 h after waking up; see Fig. 1b). If offline learning is due to training close to the night of sleep and not to the time of being awake the day after, the individuals of the G8_sleep_ should show comparable improvement with those of the G8_pm_.

**Fig. 3:**
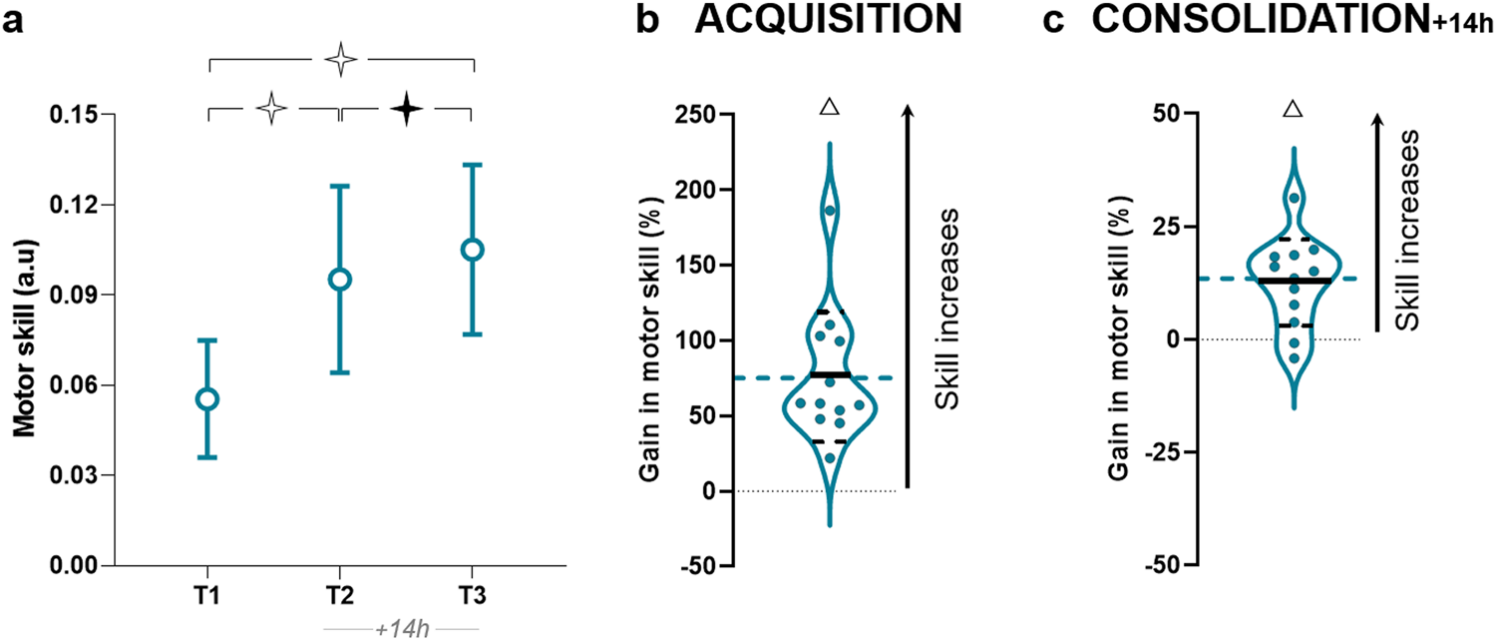
Skill performance for the G8_sleep_ group. **a** Average values (+SD) of skill in T1, T2, and T3. Repeated measures ANOVA and Newman–Keuls post hoc comparisons were applied to the data. Stars indicate significant differences between sessions. ✧ *p* < 0.001, ✦ *p* < 0.05. **b** Violin plots for the percentage of acquisition gain in skill (T1_T2). **c** Violin plots for the percentage of consolidation gain in skill (T2_T3). Thick and thin horizontal lines mark mean and SD, respectively. Dots represent individual data. Triangles indicate significant differences from the value *zero* (*T*-test). Δ *p* < 0.001. Hatched blue lines correspond to the mean of G8_pm_.

The rmANOVA revealed a significant effect of *session* for skill (F_2,22_ = 65.25, *p* < 0.001, η^2^ = 0.85; see Fig. 3a). The *post-hoc* analysis showed significant differences between all measurements (T1 versus T2: *p* < 0.001; T2 versus T3; *p* = 0.04; T1 versus T3: *p* < 0.001). No significant difference between the G8_sleep_ and the G8_pm_ was found for the acquisition gain T1_T2 (*t* = 0.07, *p* = 0.94, *d* = 0.03; Bayesian equivalence test: BF^OH^_01_ = 2.67 and BF^NOH^_01_ = 2.89; see Fig. 3b). The G8_sleep_ also significantly improved skill during the training (compared to reference value *zero* (0); *t* = 6.07, *p* < 0.001, *d* = 1.75; see Fig. 3b). After the night of sleep (T2_T3), the G8_sleep_ was not different (*t* = −0.18, *p* = 0.86, *d* = 0.07; Bayesian equivalence test: BF^OH^_01_ = 2.64 and BF^NOH^_01_ = 2.86) to that obtained for the G8_pm_. We observed a significant offline improvement (compared to reference value *zero* (0): *t* = 4.44, *p* < 0.001, *d* = 1.28; 10/12 participants increased their performance). In addition, T2_T3 gain in skill for the G8_sleep_ was significantly greater than for the group G10_am_ (*t* = 3.80, *p* < 0.001, *d* = 1.74).

According to these results, skill consolidation seems to be independent of the time one is awake the day after the *training-session*, further reinforcing the premise that sleep significantly better contributes to skill consolidation if the acquisition is performed closer to sleep time.

### Control experiment 2

To directly attribute the skill consolidation to sleep, one must also examine for any positive effect of the passage of time between training and sleep. Approximately, 4 h elapsed between the beginning of training (8 p.m.) and time of sleep (12 a.m.) for the G8_pm_, and therefore, one could not exclude any improvement/consolidation in skill performance during this period. To evaluate such an effect, the G8_awake_ carried out the training at 8 p.m. and was retested 2 h later at 10 p.m.

**Fig. 4:**
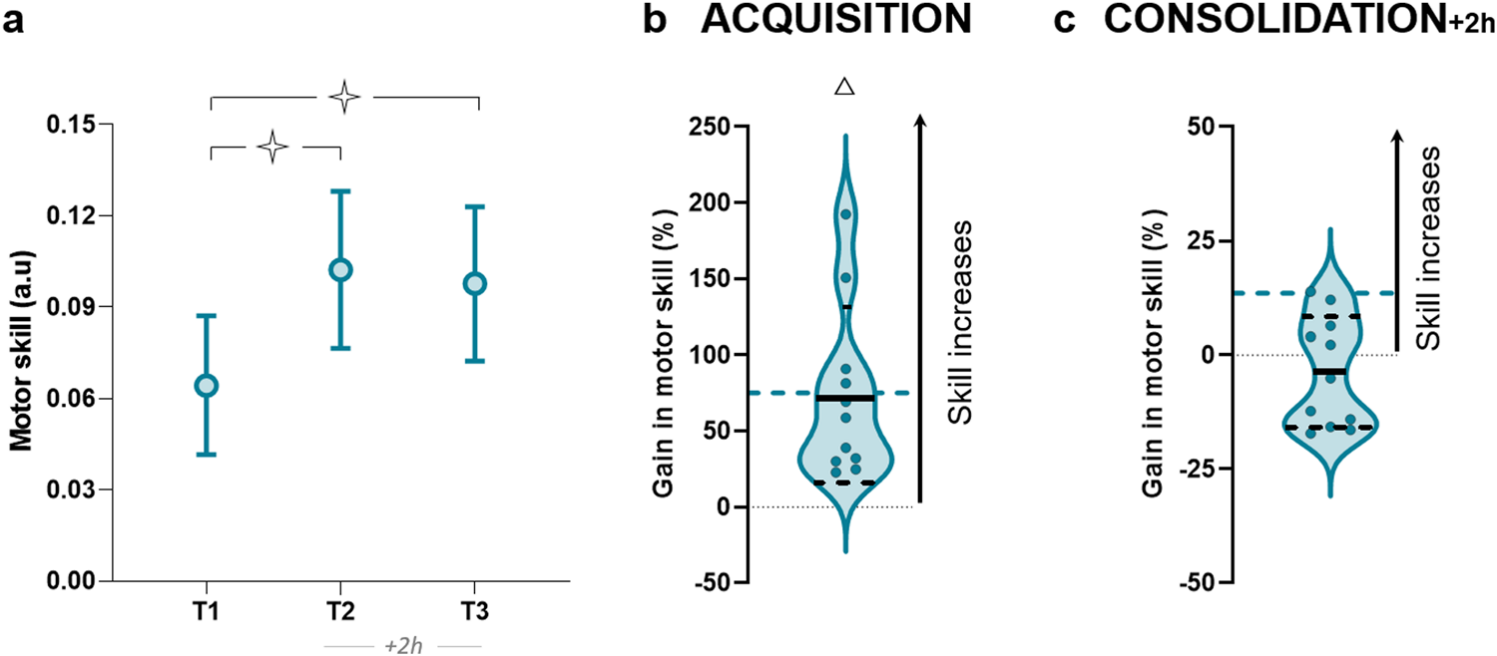
Skill performance for the G8_awake_ group. **a** Average values (+SD) of skill in T1, T2, and T3. Open stars indicate significant differences between sessions. Repeated measures ANOVA and Newman-Keuls post hoc comparisons were applied to the data. Stars indicate significant differences between sessions. ✧ *p* < 0.001. **b** Violin plots for the percentage of acquisition gain in skill (T1_T2). **c** Violin plots for the percentage of consolidation gain in skill (T2_T3). Thick and thin horizontal lines mark mean and SD, respectively. Dots represent individual data. The triangle indicates a significant difference from the value *zero* (*T*-test). Δ *p* < 0.001. Hatched blue lines correspond to the mean of G8_pm_.

Figure 4a illustrates the average values (+SD) of skill for G8_awake_. The rmANOVA revealed a significant session effect (F_2,20_ = 25.56, *p* < 0.001, η^2^ = 0.72). The *post-hoc* analysis showed a significant difference between the first session (T1) and the other sessions (T2 and T3; *p* < 0.001 for both), while no significant difference was detected between T2 and T3 (*p* = 0.44; Bayesian equivalence test: BF^OH^_01_ = 3.12 and BF^NOH^_01_ = 3.18). The acquisition gain (T1_T2) was comparable to G8_pm_ (*t* = −0.16, *p* = 0.86, *d* = 0.07; Bayesian equivalence test: BF^OH^_01_ = 2.61 and BF^NOH^_01_ = 2.82; see Fig. 4b). The G8_awake_ also significantly improved the skill during the training (compared to reference value *zero* (0): *t* = 4.34, *p* = 0.001, *d* = 1.31). After 2 h, the consolidation gain (T2_T3) of G8_awake_ was significantly inferior to the offline gain of the G8_pm_ (*t* = −2.99, p = 0.007, *d* = 1.25; see Fig. 4c). The comparison of T2_T3 gain to reference value *zero* (0) did not show improvement for G8_awake_ (*t* = −1.07, *p* = 0.31, *d* = 0.32). Overall, these results firmly support the contribution of sleep in the offline improvement of skill performance when the training is planned close to sleep.

## Discussion

In the current study, we examined the influence of the time-of-day on motor skill acquisition and consolidation on a finger-tapping task. Our findings did not show any effect of the time-of day (10 a.m., 3 p.m., and 8 p.m.) in the acquisition process (i.e., skill improvement during the training session) as all groups equally improved their skill performance. Consolidation, however, significantly differed according to the time-of-day. Precisely, we observed a deterioration in skill performance 24 h after training in the morning (G10_am_), a stabilization after training in the afternoon (G3_pm_), and an improvement after training in the evening (G8_pm_). In addition, the findings from the two control groups suggest that offline gains in skill performance for the evening group were mainly due to the night of sleep and not to the awakening time between training and sleep or the amount of time passed awake after sleep (G8_awake_ and G8_sleep_, respectively).

As in many previous findings^6,25,26,36–39^, all participants in our study increased skill performance following a single session of practice. Interestingly, the total skill improvement was comparable between the different times-of-day in which training was provided (morning, afternoon, or evening). Furthermore, the skill learning rate (see learning curves, Fig. 2c) was also similar among groups and thus according to the time-of-day. These findings indicate that skill acquisition is independent of the time-of-day in which training is administrated.

These results may be surprising since clear influences of the time-of-day on behavioral and neural processes have been described^16,18^. For instance, the maximal voluntary contraction^19^, speed/accuracy tradeoff of actual and mental movements^21^, handwriting^22^, and badminton serve accuracy^40^ fluctuate through the day. It is proposed that the naturally higher values of body temperature in the late evening compared to the morning may enhance motor performance due to hormonal and muscular adaptations^41,42^. It must be noted that in these studies, which did not evaluate the motor learning process, performances were already acquired at the moment of the tests, possibly explaining the differences with our results. In addition, neurophysiological mechanisms, such as Long-Term Potentiation (LTP-like plasticity) and intracortical inhibition within the primary motor cortex (M1), which are both involved in skill acquisition^43–46^, are also modulated by the time of day. For example, Sale et al. ^31^ showed that these neural mechanisms seem to vary across the day according to the cortisol hormonal circadian fluctuation. Furthermore, Lang et al. ^47^ showed that the inhibitory networks on M1 (notably by GABA-B mediated), influencing neural plasticity^48^, also fluctuated throughout the day.

In the present study, such plastic neural changes within M1 did not seem to affect skill acquisition. Possibly, daily fluctuations of plastic neural changes influence consolidation rather than the acquisition of motor skills. In addition, the acquisition of motor sequences is a complex process, which is not limited to the M1 area but includes different cortico-subcortical networks such as cortico-striatal (CS) and cortico-cerebellar (CC) networks^3,49^. It is also important to note that skill acquisition includes multiple mechanisms^50,51^ and reflects the sum of different learning components, such as implicit and explicit learning^1,52–54^. In the present study, we did not explore these mechanisms analytically. Isolating these components to examine their evolution across the day may provide a better understanding of our results.

While the time of day did not influence the acquisition, we discovered different outcomes concerning consolidation. A majority of studies have shown offline learning, i.e., additional improvement of performance without practice after a night of sleep^6,25,27,36,38,55–57^. Here, we found that this offline learning occurs only if the training takes place in the evening, followed by a night of sleep. More precisely, we observed different amounts of consolidation according to the training schedule, with a deterioration of motor skill for the morning group (10 a.m.), a stabilization for the afternoon group (3 p.m.), and an improvement for the evening group (8 p.m.) 24 h after the training. Notably, our results showed that the offline skill improvement of the evening group was due to a consolidation process (see Fig. 2c with the T3_pred_ and Fig. 2d with the gain of G8pred), and not due to a simple continuation of the learning^12^. In such a case, one should observe the same amount of improvement from 24 h later (T3) to the extrapolation of learning (T3_pred_). Despite restructuring our experimental protocol to reduce the potential biases^58^, a part of the supplementary improvement observed may also be attributed to the dissipation of reactive inhibition that occurs during the acquisition process^12^. However, it is important to note that this does not alter the conclusion about the different consolidations observed.

Additionally, we showed that offline learning did not occur with the simple passage of time just after training (G8_awake_; see control experiment 2) and that sleep is the key factor for off-line learning. We also found comparable offline improvement when the retest was scheduled in the morning or the evening of Day 2 (G8_sleep_ vs G8_pm_; see control experiment 1), excluding the possible contribution of the retest schedule and, thus, the time being awake after the night of sleep. Indeed, many studies have reported changes in non-rapid-eye-movement (NREM) stage-2 sleep as well as reactivations of task-related memory networks during sleep following a training session^59,60^. Moreover, it appears that sleep facilitates skill consolidation with a favorable molecular and cellular environment for plasticity^61^, which is the key process of motor learning to improve performance and memory^11,44,62^. Note that sleep quantification would undeniably add complementary information regarding the effects of sleep stages on motor consolidation in the present study. Our qualitative analysis of sleep, however, via the Pittsburgh sleep quality index, showed that all participants had good sleep quality without any differences between groups.

Overall, our findings support the specific contribution of sleep in skill consolidation when the training is administrated in the evening. Thus, there may be a temporal window after training where sleep may provide consolidation benefits. A training session close to sleep could protect against memory deterioration induced by additional memories (retroactive interferences^63,64^). The morning group, spending more time awake than the afternoon and evening groups, could be more exposed to interferences. As discussed in a previous study^30^, however, these different consolidations may result from variations in neural correlates during acquisition, which is essential for consolidation. Albouy et al. ^39^ found that the interaction between hippocampo- and striato- networks during acquisition was associated with offline gains observed during re-testing. On the other hand, Dolfen et al. ^65^ showed that increased cortisol levels through stress induction resulted in a gradual disengagement of hippocampal networks during acquisition, which was unfavorable for consolidation. Therefore, the natural fluctuation in cortisol levels, which are typically higher in the morning than in the evening^66^, could potentially harm the consolidation process. However, these explanations need to be further explored to determine whether it is the time of day or the proximity between training and sleep that influences consolidation, particularly through experimental protocols involving nap designs. For instance, it would be interesting to investigate whether taking a nap after morning training can enable offline learning.

Another hypothesis for the deterioration of the morning group may be the involvement of different memory systems during the training. The learning of a sequential finger-tapping task requires both procedural (for skill) and declarative (for knowledge) memories^67^. While procedural memory appears to consolidate during wakefulness^68,69^, the combination of procedural and declarative memories consolidate during sleep^6,25,27,36,37^. More interestingly, Brown et al. ^70^ showed a conflictual interaction between declarative and procedural memories, leading to a deterioration of performance. This conflict may be due to common cerebral structures (medial temporal lobe) or a mediating structure (the dorsolateral prefrontal cortex) being engaged in both types of memories^64,70,71^. In our study, the worst consolidation for the morning group could be due to the competition between both memories engaging in our finger-tapping task. Further investigation is needed to provide a better understanding of the underlying mechanisms contributing to the time-of-day influence on consolidation processes.

Skill consolidation according to the time of day does not appear to be population-specific but is a robust phenomenon occurring regardless of age and chronotype. Better consolidation was found after evening training in both adolescent^29^ and elderly^72^ populations. Moreover, although our study mainly included intermediate-type chronotypes (see also Supplementary notes 3 with only intermediate-type participants), we found a similar conclusion as the study of Korman et al. ^72^, which showed an advantage in consolidation when the practice was scheduled in the evening for participants with a morning-type chronotype. It should be noted that in their study the advantage in speed observed in the evening group was associated with a decrease in the time between sequences rather that the time within sequences.

Finally, the current study suggests that the timing of practice is an important factor for motor consolidation, which is better after training in the evening than in the morning. Even if new investigations are necessary to generalize these results and to better understand the underlying neural mechanisms, our findings may help in planning effective interventions in sports and rehabilitation. This conclusion applies primarily to individuals with an intermediate chronotype, as the majority of participants in the study belonged to this type. It would be interesting to expand this research to include populations with different chronotypes to broaden our recommendations.

## Methods

### Participants

Sixty healthy adults participated in the study after giving their written informed consent. One participant was excluded due to non-compliance with instruction, leading to fifty-nine participants (*n* = 59) included in the final sample. All were right-handed (mean score 0.8 ± 0.2), as defined by the Edinburgh handedness questionnaire^73^, and free from any neurological or physical disorder. Participants performed sequence typing tasks. Due to the nature of this fine motor task, we excluded musicians and professional typists. For the main experiment, thirty-six participants were randomly assigned into three groups according to when they experimented: the G10_am_ (*n* = 12, 8 females, mean age: 25 ± 6 years old), the G3_pm_ (*n* = 12, 7 females, mean age: 24 ± 6 years old), the G8_pm_ (*n* = 12, 6 females, mean age: 23 ± 2 years old). Note that, data from participants of G10_am_ and G3_pm_ were also used in a previously published study^30^. The remaining twenty-three participants were assigned into two control groups: the G8_sleep_ (*n* = 12, 5 females, mean age: 23 ± 4 years old), and the G8_awake_ (*n* = 11, 4 females, mean age: 26 ± 3 years old). The experimental design was approved by the regional ethic committee (Comité de Protection des Personnes - Région EST) and conformed to the standards set by the Declaration of Helsinki.

All participants were requested to be drug- and alcohol-free throughout the whole experiment and the night before, to not change their usual sleep (at their home) and daily activities (e.g., cooking, computer use, handicraft), and to not make intensive physical activity during the 24 h preceding the experiments. They were all synchronized with a normal diurnal activity (8 a.m. ± 1 h to 12 a.m. ± 1 h alternating with the night). We examined the chronotype of each participant using the Morningness-Eveningness Questionnaire^74^. In this test, scores range from 16 to 86 and are divided into five categories: evening type (score 16 to 30, *n* = 2 for our study), moderate evening type (score 31 to 41, *n* = 5), intermediate type (score 42 to 58, *n* = 45), moderate morning type (score 59 to 69, *n* = 6), and morning type (score 70 to 86, *n* = 1). There were no significant differences between groups regarding the chronotype (on average; G10_am_: 51 ± 11; G3_pm_: 50 ± 9; G8_pm_: 52 ± 10; G8_awake_: 54 ± 10; G8_sleep_: 47 ± 8; one-way ANOVA: F_4,54_ = 1.05, *p* = 0.39, η^2^ = 0.06). We verified the sleep quality of each participant with the Pittsburgh Sleep Quality Index^75^. The general score in this questionnaire ranges from 0 (no particular difficulties sleeping) to 21 (major difficulties sleeping). The results indicated good sleep quality for all groups (on average; G10_am_: 5 ± 1, G3_pm_: 4 ± 1, G8_pm_: 5 ± 3; G8_awake_: 4 ± 3; G8_sleep_: 5 ± 3; one-way ANOVA: F_4,54_ = 0.28, *p* = 0.89, η^2^ = 0.03).

### Experimental device and procedure

Participants comfortably sat on a chair in front of a keyboard. We employed a computerized version of the sequential finger-tapping task^30^. Specifically, participants were requested to tap as accurately and as fast as possible with their non-dominant hands in the following sequence: 1-4-2-3-1-0. Each key was affected by a specific finger: 0-thumb, 1-index, 2-middle, 3-ring, and 4-little. One trial was composed of six sequences performed in a row. At the beginning of each trial, participants pressed the key ‘0’ with their thumb to start the chronometer and they accomplished the 6 sequences continuously. Pressing the key ‘0’ at the end of the 6^th^ sequence stopped the chronometer and ended the trial (Fig. 1a).

The experiments included two *sessions*, scheduled on two consecutive days (Day 1 and Day 2), except for the G8_awake_ (tested only on Day 1, see Fig. 1b). On Day 1, participants were tested at 10 a.m. (G10_am_), 3 p.m. (G3_pm_) or 8 p.m. (G8_pm_, G8_awake_, and G8_sleep_). They carried out 48 trials (12 blocks of 4 trials, with 5-s rest between trials and 30-s rest between blocks). The score in the first two trials (1 and 2) and the last two trials (47 and 48) constituted the pre-test (T1) and the post-test (T2), respectively. The remaining trials (*n* = 44; from the 3^rd^ to the 46^th^ trial) constituted the training trials. To familiarize themselves with the protocol, all participants accomplished two trials at a natural speed. For the *retest-session* (consolidation), all participants carried out two trials (T3). The G10_am_, G3_pm,_ and G8_pm_ were re-tested 24 hours (Day 2) after the first *training-session*.

When testing skill consolidation following different training times, one is faced with a double problem: the effect of the passage of time and the effect of sleep, since both could contribute to motor consolidation^68,76^. For that reason, we included two supplementary groups to specifically test whether the time being awake before and after the night of sleep affects skill consolidation. The G8_sleep_ was re-tested 14 h (Day 2 at 10 a.m.) after the first *training-session*. The G8_awake_ was re-tested 2 h (Day 1 at 10 p.m.) after the first *training-session*. If only the night of sleep contributes to the development of off-line learning, we should not observe changes in skill performance before sleep (G8_awake_), while offline gains should be observed immediately after sleep (G8_sleep_).

The vision of the non-dominant hand was hidden using a box during the whole protocol. The sequence’s order, however, was displayed on the box and thus visible to the participants during the whole experiment. No information concerning motor performance (i.e., time or typing errors) was provided to the participants.

### Data recording and analysis

For each trial, movement accuracy and duration were computed. The accuracy (‘Error rate’) was defined as the number of false sequences throughout one trial (0 = no false trial; 6 = all trials false). If the participants made one or more mistake(s) in one of the sequences, this sequence was counted as false (see Fig. 1a). The error rate was defined as the percentage of the number of errors during a trial:

1 $${Error}\,{rate}=\,\frac{{nb}\,{of}\,{errors}}{6}\,\times \,100$$

Movement duration was defined as the time interval (in seconds) between the start of the trial (when the participant pressed the key ‘0’) and the end of the trial (when the participant pressed the key ‘0’ at the end of the 6^th^ sequence).

These two parameters (Movement duration and Error rate) are related by the speed-accuracy tradeoff function^77^. Ascertaining, thus, that motor skill (i.e., the training-related change in the speed-accuracy trade-off function) has been improved is not possible when duration and accuracy change in opposite directions. For that reason, we compute a composite ratio (a.u) to describe motor skills as follows:

2 $${Skill}=\,\frac{1-(\frac{{nb}\,{of}\,{errors}}{6})}{{duration}}$$

In that formula, skill increases if the duration decreases and/or if the number of errors decreases.

Gains between sessions were calculated following a simple proportional formula:

3 $${\rm{Gain}}\; {\rm{T}}1{\_}{\rm{T}}2=\frac{{\rm{T}}2-{\rm{T}}1}{{\rm{T}}1}{\times} 100{{;}}\,{it}\,{indicates}\,{the}\,{amount}\,{of}\,{skill}\,{acquisition}$$

4 $${\rm{Gain}}\; {\rm{T}}2{\_}{\rm{T}}3=\frac{{\rm{T}}3-{\rm{T}}2}{{\rm{T}}2}\times 100{;}\,{it}\,{indicates}\,{the}\,{amount}\,{of}\,{skill}\,{consolidation}$$

### Statistical analysis

Based on a previous study^30^ using the same experimental design, an a priori G*POWER analysis for total sample size estimation (parameters: Effect size f = 0.30; α = 0.05; power = 0.90; groups = 3; number of measurements = 3; correlation among the repeated measures = 0.5), indicated 8 participants per group (*n* = 33). While this analysis indicated that twelve (*n* = 12) participants per group were sufficient for our study, we are aware that increasing the sample size would yield more reliable and robust results.

For all data, we verified the normality and sphericity using Shapiro–Wilk test and Mauchly’s test, respectively. For all statistical analyses, the significance level was fixed at 0.05 and the observed power was superior to 0.8.

First, we compared the G10_am_, G3_pm_, and G8_pm_. For skill, we applied repeated measures (rm) ANOVA with *group* as between-subjects factor (G10_am_, G3_pm_, and G8_pm_) and *session* as within-subjects factor (T1, T2, and T3). Gains in motor skills (T1_T2 and T2_T3) were analyzed by one-way ANOVA between groups. When necessary, the *post-hoc* analyses were performed by applying Newman-Keuls tests. Moreover, each gain was compared with the reference value *zero* (T-test).

We performed the same analyses on the movement duration (see Supplementary notes 1). As the error rate did not follow a normal distribution (Shapiro–Wilk test, *p* < 0.05), we used two-tailed permutation tests (5000 permutations; MATLAB function mult_comp_perm_t1). *P*-values were corrected for multiple comparisons using the Benjamini-Hochberg False Discovery Rate (MATLAB function fdr_bh) (see Supplementary notes 1).

In addition, the trial-by-trial (*n* = 48) evolution of individual skill performance during the whole training session was analyzed by a power-law function:

5 $${Skill}=a.{{Trial}}^{b}$$

where *a* is the learning gain and the exponential *b* is the learning rate. We used one-way ANOVA between groups for the learning rate (*b*). To further analyze the form of each learning curve (represented by the power-law function), we determined the moment at which the skill performance reached an asymptote. Precisely, we computed the derivative of the fitted power function for each participant. To define the moment at which skill progression tends to plateau, we computed the time point at which the derivative (i.e., the difference between skill at t1 + 1 and skill at T1) reached 0.0001 above its minimum value for each participant. We compared these values between groups using a one-way ANOVA. Since our threshold was arbitrarily determined, we performed, as a control, the same analysis with threshold values of 0.0002, 0.0003, 0.0004, and 0.0005 (results of these analyses are presented in Supplementary notes 2).

To test whether a possible improvement in skill performance at T3 was due to offline learning rather than the continuation of training^58^, we extrapolated the individual skill performance during the 48 trials to an additional two trials (49 and 50) in G8_pm_. For each participant, we determined the gain T2_T3_pred_ based on the post-test (T2) and the individual predicted evolution of training (T3_pred_) and compared it with the gain T2_T3 of G8_pm_ by independent samples T-test.

We also tested the skill of G8_sleep_ using rmANOVA with the *session* as within-subject factor (T1, T2, and T3) and performed Newman-Keuls post hoc. As in the first analysis, the skill gains (T1_T2 and T2_T3) of G8_sleep_ were compared to the reference value ‘*zero’* (0). In addition, we compared skill gains of G8_sleep_ with G8_pm_ with independent t-tests. Finally, we performed analogous statistical analyses for the G8_awake_. In addition, the movement duration and the error rate were analyzed (see Supplementary notes 1).

The effect size of ANOVA is reported as partial eta squared (η^2^) with small (≥0.01), moderate (≥0.07), and large effects (≥0.14). The effect sizes (Cohen’s *d* with small, moderate, and large effects, respectively as ≥0.20, ≥0.50, and ≥0.80) were also reported for *t*-tests. When necessary, to provide additional support for statistical analyses when null effects were obtained, Bayesian equivalence analysis was performed with a region of practical equivalence ROPE = [−0.1,0.1] and a prior Cauchy scale of 0.707^78^. This type of analysis quantifies the evidence in favor of the null hypothesis and discriminates the ‘absence of evidence’ and the ‘evidence of absence’, leading to stronger and more reliable conclusions.

### Reporting summary

Further information on research design is available in the Nature Research Reporting Summary linked to this article.

### Supplementary information

Supplementary materials

Reporting summary

## Data Availability

All data from this study are available at: https://osf.io/nzd92/?view_only=7d1038c96bd04f808282a304793dd03a.
