# Supplementary material for: Time of day and sleep effects on motor acquisition and consolidation
Source: NPJ Sci Learn. 2023 Sep 1;8:30. doi: 10.1038/s41539-023-00176-9 (PMC10474136; doi:10.1038/s41539-023-00176-9)
Supplement: Supplementary file 1 — Supplementary materials [file 41539_2023_176_MOESM1_ESM.pdf]

# Supplementary materials

## Supplementary notes 1

### Main experiment

Supplementary Figure 1 illustrates the average (+SD) of duration and error rates for $G10_{am}$, $G3_{pm,}$ and $G8_{pm}$. Analyses for duration (rmANOVA *group* x *session*: $F_{4,66} = 2.61$, $p = 0.04$, $\eta^2 = 0.14$; *post-hoc* analysis: $p > 0.58$ in all cases) and accuracy (permutation tests: $T < 0.35$, $p > 0.97$ in all cases) did not show any group effect on T1 either.

For the acquisition, skill improvement between T1 and T2 was mainly due to faster movements (*post hoc* analysis: $p < 0.001$ in all cases) rather than more accurate movements (permutation tests: $T < 1.99$, $p > 0.12$ in all cases). Moreover, for the consolidation, analyses did not reveal any statistical difference between T2 and T3 either for the duration (post hoc analysis: $p > 0.20$ in all cases) or accuracy (permutation tests: $T < 1.94$, $p > 0.09$ in all cases), suggesting that changes in skill consolidation were due to combined fine changes in both duration and accuracy.

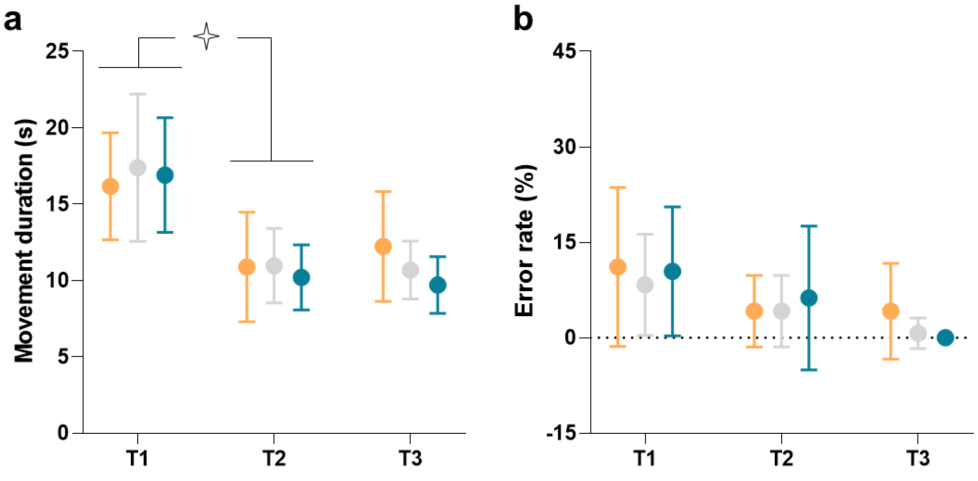


**Supplementary Figure 1.** Supplementary Results. **(a)** Average values and standard deviations (+SD) of movement duration in T1, T2, and T3 for the $G10_{am}$, $G3_{pm}$, and $G8_{pm}$. A repeated measures ANOVA and Newman-Keuls post hoc comparison were applied to the data. The star indicates significant differences between sessions. ✧ : $p < 0.001$. **(b)** Average values and standard deviations (+SD) of error rates in T1, T2, and T3 for the $G10_{am}$, $G3_{pm}$, and $G8_{pm}$

No significant difference was found by using two-tailed permutation tests between sessions and groups with the Benjamini-Hochberg False Discovery Rate correction.

## Control 1

Duration and accuracy of $G8_{sleep}$ (see Supplementary Figure 2) contributed in the same way as $G8_{pm}$ for the skill acquisition, i.e., improvement in duration (T1 versus T2; $F_{2,22} = 66.87$, $p < 0.001$, $\eta^2 = 0.86$; *post hoc* analysis: $p < 0.001$) but not in accuracy (permutation test: T = 1.72, p = 0.21), and consolidation, i.e., slight but non-significant improvement, for both duration and accuracy (T2 versus T3; for movement duration: *post hoc* analysis: p = 0.16; for error rate: T = 1.12, p = 0.57).

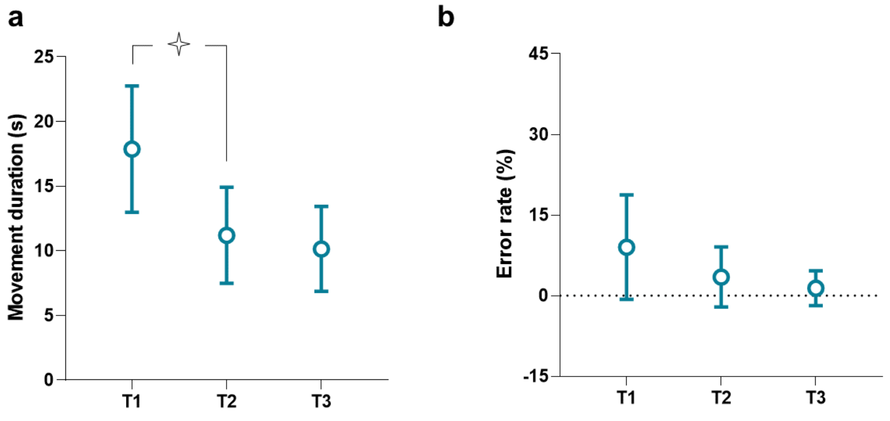


**Supplementary Figure 2.** Supplementary Results. **(a)** Average values and standard deviations (+SD) of movement duration in T1, T2, and T3 for the $G8_{sleep}$. A repeated measures ANOVA and Newman-Keuls post hoc comparisons were applied to the data. The star indicates significant differences between sessions. ✧ : $p < 0.001$. **(b)** Average values and standard deviations (+SD) of error rates in T1, T2, and T3 for $G8_{sleep}$. No significant difference was found by using two-tailed permutation tests between sessions with the Benjamini-Hochberg False Discovery Rate correction.

## Control 2

Like the previous group, the duration and accuracy of $G8_{awake}$ (see Supplementary Figure 3) contributed in the same way as $G8_{pm}$ for the skill acquisition (T1 versus T2; significant

improvement for duration: $F_{2,20} = 15.23$, $p < 0.001$, $\eta^2 = 0.60$; *post hoc* analysis: $p < 0.001$; no significant improvement for accuracy; permutation test: T = 1.51, p = 0.53). However, for the consolidation, we observed slight deterioration, but no significance, of both duration (T2 versus T3; *post hoc* analysis: p = 0.82) and accuracy (permutation test: T = 0.77, p = 0.66).

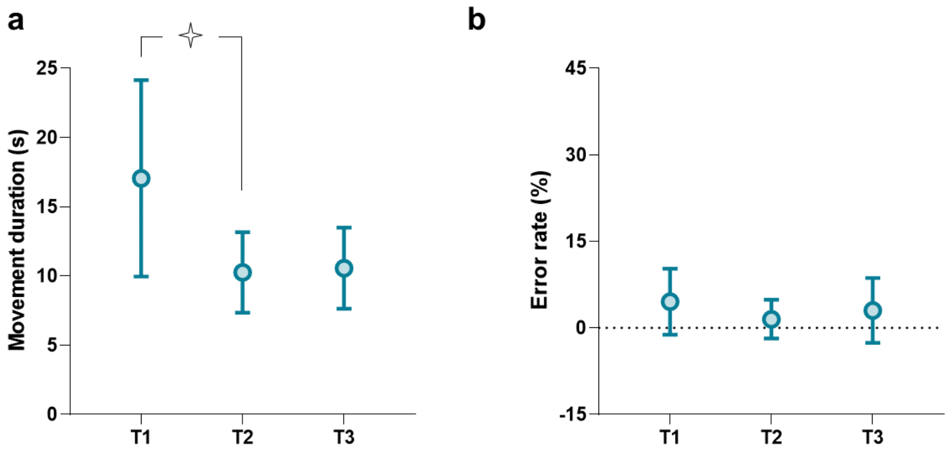


**Supplementary Figure 3.** Supplementary Results. **(a)** Average values and standard deviations (+SD) of movement duration in T1, T2, and T3 for the $G8_{awake}$. A repeated measures ANOVA and Newman-Keuls post hoc comparisons were applied to the data. The star indicates significant differences between sessions. ✧ : $p < 0.001$. **(b)** Average values and standard deviations (+SD) of error rates in T1, T2, and T3 for $G8_{awake}$. No significant difference was found by using two-tailed permutation tests between sessions with the Benjamini-Hochberg False Discovery Rate correction.

## Supplementary notes 2

### Threshold values for the plateau performance

Since our threshold value of 0.001 was arbitrarily determined, we performed, as a control, the same analysis with a threshold value of 0.0002, 0.0003, 0.0004, and 0.0005. Table S1 indicates the mean (+SD) of the trial when groups reached the performance plateau according to the four different threshold values. One-way ANOVA did not find significant differences between groups, whatever the threshold values chosen (with 0.0002: $F_{2,33} = 0.18$, $p = 0.83$, $\eta^2 = 0.01$; with 0.0003: $F_{2,33} = 0.11$, $p = 0.90$, $\eta^2 = 0.01$; with 0.0004: $F_{2,33} = 0.22$, $p = 0.80$, $\eta^2 = 0.01$; with 0.0005: $F_{2,33} = 0.05$, $p = 0.95$, $\eta^2 = 0.01$).

**Tableau S2.** Supplementary Results. The mean (+SD) of the first trial of the performance plateau according to the different threshold values for each group.

| Groups | | First trial of the performance plateau | | | |
|---|---|---|---|---|---|
| | | **0.0002** | **0.0003** | **0.0004** | **0.0005** |
| **$G10_{am}$** | Mean | 18 | 14 | 10 | 8 |
| | *SD* | *5* | *5* | *4* | *4* |
| **$G3_{pm}$** | Mean | 19 | 15 | 11 | 9 |
| | *SD* | *3* | *3* | *3* | *3* |
| **$G8_{pm}$** | Mean | 19 | 14 | 11 | 8 |
| | *SD* | *4* | *4* | *3* | *3* |

## Supplementary notes 3

### The $G10_{am}$, $G3_{pm}$, and $G8_{pm}$ groups without extreme and moderate chronotypes

Supplementary Figure 4a shows the average values (SD) of skill after excluding 9 participants from the statistical analysis, 3 from each group with extreme and moderate chronotypes. rmANOVA revealed a significant interaction effect for skill ($F_{4,48} = 5.81$; $p < 0.001$, $\eta^2 = 0.33$). The post-hoc analysis did not show significant differences between groups in T1 ($p > 0.80$; Bayesian equivalence tests: $BF^{OH}_{01} > 2.27$ and $BF^{NOH}_{01} > 2.30$ in all cases).

Skill significantly enhanced after training for all groups (T1 versus T2; $p < 0.001$ in all cases). Supplementary Figure 4b illustrates the average (+SD) acquisition gains (T1_T2) in skill. The comparison of T1_T2 gain with the reference value *zero* (0) showed significant improvement in skill for all groups (in all, $t > 4.74$, $p < 0.01$, $d < 0.28$; see Supplementary Figure 4b); this improvement was similar between groups (one-way ANOVA: $F_{2,24} = 0.19$, $p = 0.82$, $\eta^2 = 0{,}02$; Bayesian equivalence tests: $BF^{OH}_{01} > 2.14$ and $BF^{NOH}_{01} > 2.17$ in all cases).

One day after training (T2 vs T3; *post hoc* analysis), we observed a deterioration in skill performance for the $G10_{am}$ ($p = 0.009$), a stabilization for the $G3_{pm}$ ($p = 0.46$), and further improvement for the $G8_{pm}$ ($p = 0.02$). Supplementary Figure 4c illustrates the average (+SD) consolidation gains (T2_T3). One-way ANOVA ($F_{2,24} = 9.25$, $p = 0.001$, $\eta^2 = 0{,}44$) showed that

the T2_T3 gain of the $G10_{am}$ significantly differed from that of the $G3_{pm}$ and $G8_{pm}$ (*post hoc* analysis: $p < 0.01$ for both). The T2_T3 gain compared with the reference value zero (0) showed a deterioration of skill for the $G10_{am}$ ($t = -3.37$, $p = 0.01$, $d = 1.67$; 8/9 participants decreased their performance), a stabilization for the $G3_{pm}$ ($t = 1.12$, $p = 0.30$, $d = 1.56$; 6/9 participants increased their performance) and an improvement for the $G8_{pm}$ ($t = 2.70$, $p = 0.03$, $d = 2.90$; 9/9 participants increased their performance).

All groups acquired better skill performance one-day latter (T3) compared to their initial performance (T1 versus T3; *post hoc* analysis: $p < 0.001$ in all cases).

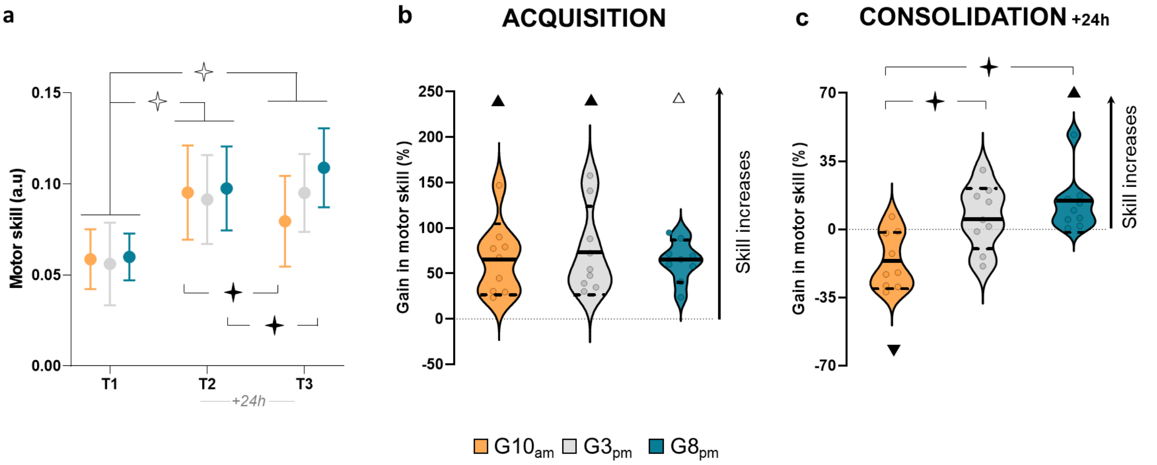


**Figure Supplementary 4.** Skill performance for the $G10_{am}$, $G3_{pm}$, and $G8_{pm}$ groups without extreme and moderate chronotype. **(a)** Average values and standard deviations (+SD) of skill in T1, T2, and T3 for each group. A repeated measures ANOVA and Newman-Keuls post hoc comparisons were applied to the data. **(b)** Violin plots for the percentage of acquisition gain in skill (T1_T2). Thick and thin horizontal lines mark mean and SD, respectively. Dots represent individual data. No significant difference was found with a one-way ANOVA between groups. **(c)** Violin plots for the percentage of consolidation gain in skill (T2_T3) for each group. Thick and thin horizontal lines mark mean and SD, respectively. Dots represent individual data. A one-way ANOVA and Newman-Keuls post hoc comparisons were applied between $G10_{am}$, $G3_{pm}$, and $G8_{pm}$. Stars indicate significant differences between groups or sessions. ✧ : $p < 0.001$, ✦ : $p < 0.05$. Triangles indicate significant differences from the value zero (T-test). △ : $p < 0.001$, ▲ : $p < 0.05$.
